# Communication barriers in perinatal care for autistic women: A qualitative study using the hermeneutic-dialectic circle in Brazil

**DOI:** 10.1371/journal.pone.0358429

**Published:** 2026-09-16

**Authors:** Aline Veras Morais Brilhante, Christina César Praça Brasil, Raimunda Magalhães da Silva, Marjan Askari, Luciana Andrade da Mota Sampaio, Jonas Loiola Gonçalves, Layane Sabóia, Lucas Alessandro Macedo Cruz, Helena Rodrigues Dias, Luciel Almeida Cavalcanti, Maria Julya Albuquerque Parente, Mariana Tavares Rocha, Melania Maria Ramos de Amorim

**Affiliations:** 1 Department of Women’s, Children’s and Adolescent Health, Faculty of Medicine, Federal University of Ceará (UFC), Fortaleza, Ceará, Brazil; 2 Graduate Program in Collective Health, University of Fortaleza (UNIFOR), Fortaleza, Ceará, Brazil; 3 Leiden University Medical Center (LUMC), Leiden, South Holland, The Netherlands; 4 Department of Neurology, School of Medicine, University of São Paulo, São Paulo, Brazil; 5 Professor Fernando Figueira Institute of Integral Medicine (IMIP), Recife, Brazil; Father Muller Charitable Institutions, INDIA

## Abstract

Autistic women experience increased obstetric and perinatal risks, partly driven by communication barriers within maternity care. Evidence on how these barriers are experienced in low- and middle-income countries remains scarce. This study aimed to analyze communication barriers in perinatal care based on narratives of autistic women who experienced pregnancy, childbirth, and postpartum care in Brazil. A qualitative study was conducted using the Hermeneutic-Dialectic Circle (HDC) approach. Thirty-four autistic women (22 with support level 1 and 12 with support level 2, including three non-speaking participants with complex communication needs) were recruited through convenience sampling, supplemented by active recruitment through participant referral, and participated in semi-structured interviews conducted in multiple accessible formats (video call, real-time messaging, in-person, and augmentative and alternative communication). Data were analyzed using constant comparative methods within the hermeneutic-dialectic framework, guided by the social model of disability and the neurodiversity paradigm. Three central categories were constructed: (1) communicational demands of autistic women and their non-recognition, including alexithymia, sensory hypersensitivity, and need for predictability; (2) institutional and interpersonal communication failures, encompassing symptom invalidation, disrespectful care, and exclusion of non-speaking women; and (3) experiences of respectful and effective communication, demonstrating that adapted strategies promote safety and autonomy. Participants’ narratives indicate that communication barriers in perinatal care for autistic women operate systemically rather than individually. Training healthcare professionals and implementing accessible communication strategies are essential to promote respectful, safe, and inclusive maternity care.

## Introduction

Recent studies demonstrate that autistic women have a higher prevalence of adverse obstetric outcomes [1–3], such as preterm birth, preeclampsia, and perinatal depression. The etiology of this disparity is multifactorial; however, communication failures during care represent a significant contributing factor [4–6]. Communication is a cornerstone of safe obstetric care: communication lapses are a primary cause of adverse events [6,7], while effective communication is associated with improved maternal and perinatal outcomes [8–10].

In autism, communication takes on particular complexity. From the perspective of the social model of disability and the neurodiversity paradigm, the analytical focus shifts from individual deficit to the inadequacy of the environment [11]. The “double empathy problem” proposes that communication difficulties between autistic and non-autistic individuals arise from a breakdown in mutual reciprocity, resulting from distinct neurological processing styles rather than from a unilateral limitation [12,13]. Under this framework, the responsibility for effective communication does not rest solely with the patient, but with the capacity of the healthcare system to adapt to the communicational needs of each individual [14,15].

Underdiagnosis and late diagnosis of autism in women further compound this scenario. It is estimated that nearly 80% of autistic women remain undiagnosed by the age of 18 [16,17], meaning that a substantial number of pregnant women may face communication barriers without any support or recognition of their needs.

Most of the available evidence on the perinatal experiences of autistic people derives from high-income countries [5,6,14,15], and its transferability to low- and middle-income settings cannot be assumed. In Brazil, maternity care is delivered through a universal public system (the Unified Health System, SUS) that coexists with a private sector, producing stratified care trajectories, and disrespect and abuse during childbirth are well documented [18]. In parallel, the underdiagnosis of autism in women [16,17] means that many autistic women navigate pregnancy without formal recognition of their needs. These structural characteristics may configure communication barriers in ways not captured by studies conducted in high-income contexts.

This study aims to analyze communication barriers in perinatal care based on the narratives of autistic women who experienced the pregnancy-postpartum cycle in Brazil. The research is justified by the scarcity of qualitative studies on this topic in low- and middle-income countries, and by its potential to identify overlooked barriers and communication strategies that may inform the development of more inclusive and safer obstetric care.

## Materials and methods

### Study design and theoretical-methodological framework

This is a qualitative study guided by the Hermeneutic-Dialectic Circle (HDC), as described by Lincoln and Guba [19]. The HDC operates through iterative cycles of understanding, interpretation, and reconstruction, producing joint constructions from the dialectical confrontation between individual narratives, empirical findings, and the literature. The approach assumes the existence of multiple socially constructed realities and seeks to develop consensus, understood not as agreement but as the identification of shared belief systems, even when anchor points and objectivations diverge [19].

As an interpretive lens, the social model of disability and the neurodiversity paradigm were adopted [13,20–22]. These frameworks propose an approach centered on maximizing abilities, offering socioemotional support, and developing strategies to address individual and environmental demands, rather than seeking to normalize conditions that constitute expressions of human diversity [23].

### Participants and recruitment

Participants were recruited through convenience sampling, supplemented by active recruitment via participant referral, from August 2024 to July 2025. The invitation was disseminated on social media platforms (Facebook, Instagram) and in WhatsApp groups of autism associations and collectives.

Inclusion criteria were: being 18 years of age or older; having a formal diagnosis of Autism Spectrum Disorder (ASD); and having experienced at least one pregnancy and delivery. Eligibility was anchored in the lived experience of gestation and childbirth: any person who had gestated and given birth could volunteer, regardless of gender identity, which encompasses cisgender women, transgender men, and non-binary persons assigned female at birth. All volunteers who enrolled were cisgender women, and the study is therefore reported with reference to autistic women. No exclusion criteria were established, with the aim of maximizing sample diversity.

The research adopted the DSM-5-TR support level classification (1, 2, and 3) [24], recognizing that this classification does not equate to severity gradations (“mild,” “moderate,” or “severe”). Classifying autism as “mild” may carry the mistaken assumption of an absence of support needs, while “severe autism” may reinforce presumptions of incapacity.

**Fig 1:**
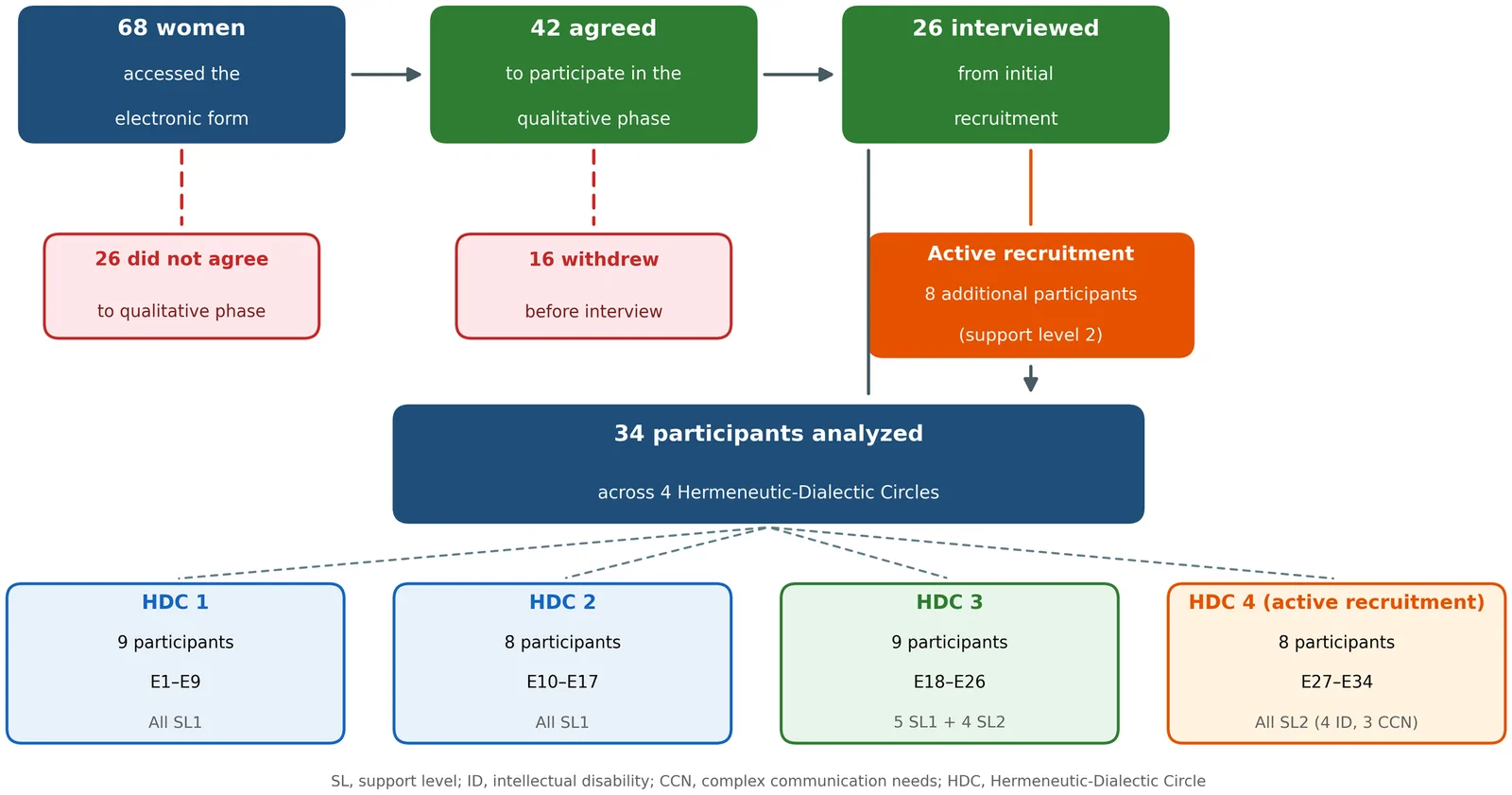
Participant recruitment and enrollment flowchart.

Initially, 68 women accessed the electronic form and completed sociodemographic data and statements of intent to participate in the qualitative phase. Of these, 42 agreed to participate, but 16 withdrew before the interview. Since the initial volunteers were predominantly at support level 1, active recruitment was conducted via referrals from previous participants, resulting in eight additional participants at support level 2, for a total of 34 participants (Fig 1).

### Data collection

Semi-structured interviews were conducted from August 2024 to September 2025. To ensure accessibility, multiple formats were offered: 15 via video call (Google Meet), 6 via real-time audio and text messaging (WhatsApp), and 13 in person (at a location chosen by the participant). This flexibility was a strategy to include women from different Brazilian states and participants with greater communicational support needs, including three non-speaking participants who communicated through augmentative and alternative communication (AAC). In three interviews with support level 2 participants with intellectual disability or complex communication needs, a facilitator was present: the legal guardian in one interview and, in two, therapeutic companions who assisted in the application of AAC. Messaging interviews were conducted via an institutional account used exclusively for research purposes. Each participant was identified by a coded name on the device. Immediately after each interview, the data were transferred to a secure file and the conversation was deleted from the device. Participants were informed of residual risks and instructed to converse in a private location and to delete the conversation after confirming receipt by the researcher.

A single researcher conducted all interviews: an obstetrician, doctoral-level physician, and university professor affiliated with a graduate program, with experience in qualitative research with autistic individuals [25,26] and a neurodivergent woman. This condition facilitated access to more restricted groups within the autistic community. The researcher had no prior personal or professional relationship with any of the participants. At the beginning of each meeting, the interviewer introduced herself, declaring her status as a physician, researcher, and neurodivergent woman, and clarifying the research motivations, a factor that proved to facilitate the establishment of rapport and an environment of mutual trust. To mitigate biases related to the researcher’s positionality, field notes included the construction of a reflexive journal, in which feelings, insights, and reflections were recorded throughout the entire process.

The interview guide was developed based on the literature and included questions about the experience of pregnancy, childbirth, and the postpartum period, with a focus on the relationship and communication with healthcare professionals. The guide was piloted with four autistic women who formed an advisory committee, following a strategy previously employed by the research group in earlier studies [25]. These four women were not part of the sample for the present study; they were external to this study, having participated in another study conducted by the principal researcher, and were specifically invited for the initial piloting of the instrument. The guide was adapted for each interview, as proposed by the HDC and in accordance with the specific communication needs of each participant [19].

Interviews had a mean duration of 40 minutes (minimum 32, maximum 63 minutes), were recorded in audio and/or video (with consent), and fully transcribed. Nine participants were contacted a second time for additional clarification. The interviews, the syntheses generated at each cycle, the field journal, and the reflexive journal comprised the analytical corpus.

### Data analysis

**Fig 2:**
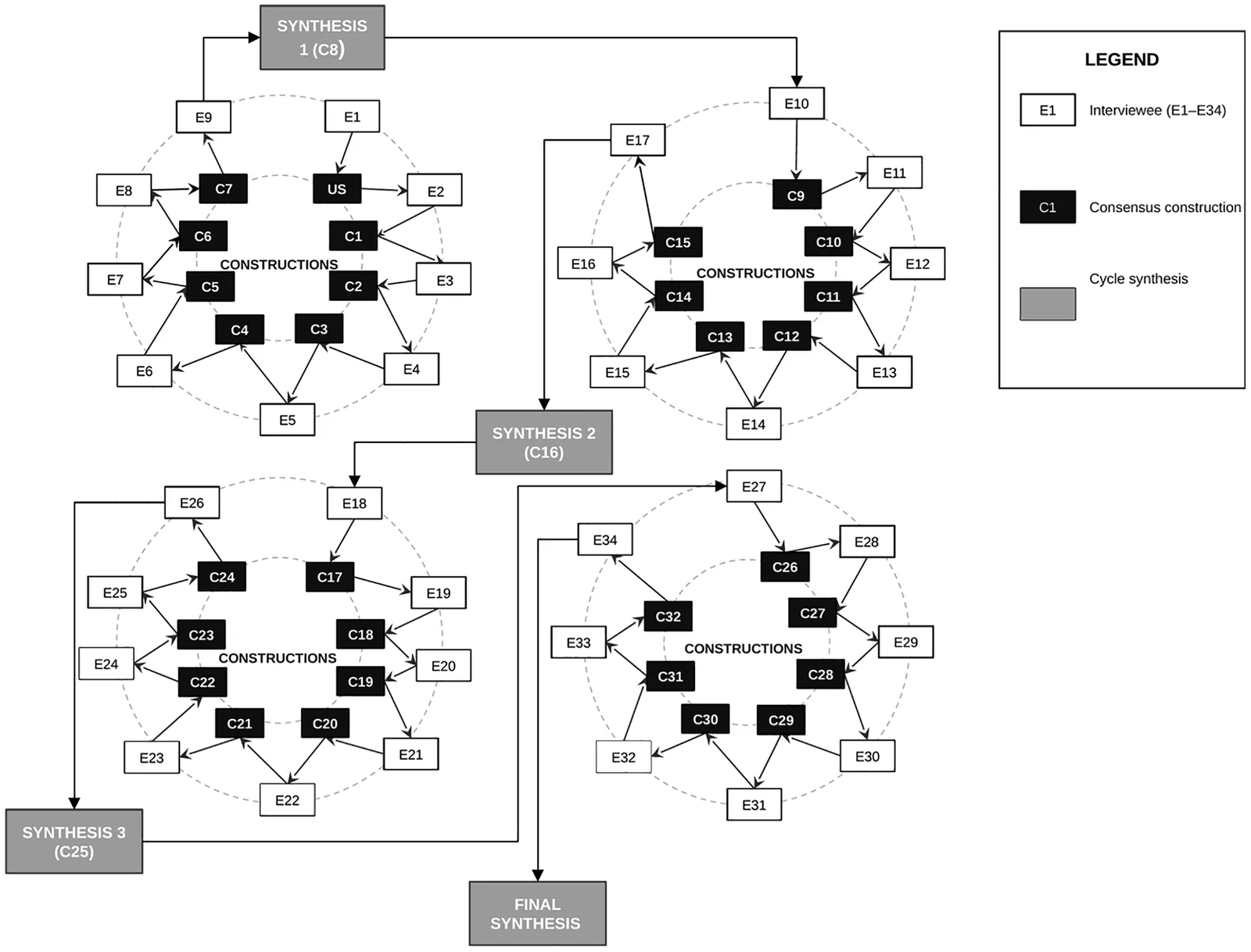
Graphical diagram of the hermeneutic-dialectic circles.

Analysis was conducted concurrently with data collection, across the four HDCs (Fig 2), using the constant comparative method [27] as the operational analytical strategy within the iterative logic of the HDC. The process involved exhaustive reading of transcripts, identification of meaning units (MUs), and initial coding, supported by NVivo software (version 12) for management and coding. Prior to analysis, the principal researcher trained two analysts in the use of NVivo and in the coding process. A randomly selected transcript from the piloting interviews was used for coding training and development of the initial codebook.

Following this preparatory process, the first interview was read independently by two researchers, who identified statements corresponding to meaning units. Subsequently, a consensus meeting was held between the two analysts and a third researcher, who mediated the discussion to consolidate the final coding and generate the codebook.

Coding disagreements were addressed, in the first instance, within these consensus meetings, mediated by the third researcher. Disagreements or interpretive uncertainties that persisted beyond the consensus meetings were taken to periodic methodological oversight meetings involving two research groups linked to two projects coordinated by the same senior researcher. In these meetings, each group presented its ongoing analysis and findings to the other group and responded to systematic questioning, functioning as a structured form of peer debriefing and external methodological scrutiny [19]. An audit trail was maintained throughout the study, comprising the interview transcripts, the syntheses and consensuses produced at each cycle, successive versions of the codebook, the field journal, and the reflexive journal.

Data generated through the different interview modalities were integrated into a single analytical corpus. Video-call and in-person interviews were transcribed verbatim; text-based interviews entered the corpus in the participants’ own written form; and AAC-mediated interviews were documented through the participants’ written productions and pictogram selections, complemented by field-journal records of contextual and non-verbal aspects. Differences in depth, spontaneity, and opportunities for non-verbal observation across modalities were monitored reflexively and explicitly considered during the consensus meetings.

The synthesis of the first interview generated the initial MUs. In the second interview, in addition to the study’s trigger questions, interpretive and evaluative questions related to the MUs emerging from the first interview were included. With each new interview, the analytical process was repeated, with progressive expansion of the codes. Due to the dialectical movement of confrontation with previous MUs, the second interview produced a more elaborate construction than the initial MUs, generating the first consensus (C1). At each cycle, the most recent consensus guided the questions posed at the end of the subsequent interview. At the conclusion of the first HDC (9 participants), a synthesis and the eighth consensus (C8) were produced.

Analytical rigor was sustained by criteria consistent with the constructivist paradigm [19]: the transparency of the interpretive process, documented in the audit trail; the researcher’s reflexivity, recorded in the reflexive journal; the dialectical confrontation of constructions at each cycle; independent coding followed by consensus meetings; peer debriefing between the research groups; and validation of the findings with participants.

Codes were grouped by semantic similarity. This grouping also occurred independently by both analysts, followed by a consensus meeting among the three researchers. Fourteen meaning clusters were identified (understood here as groupings of meaning units that express recurring interpretive patterns) and organized, ultimately, into three central categories. The final coding tree, with the categories, meaning clusters, and their operational definitions, is provided as Supporting Information (S3 Table).

### Sample adequacy

Sample adequacy was monitored using an operational saturation criterion, employing a saturation matrix and a cumulative category curve [28,29] (Table 1). After analysis of the HDC1 synthesis, the research team did not consider saturation to have been reached, and HDC2 was conducted. Although saturation could have been considered achieved after the second circle, the exclusive presence of women at support level 1 with established professional activities excluded the most vulnerable women from the analysis. Accordingly, HDC3 was conducted. After the third circle, women with greater support needs, with intellectual disabilities, or with complex communication needs were recruited, forming HDC4 (Fig 2).

**Table 1:** Saturation matrix of meaning clusters by interview.

Cycle	Interview	New Meaning Clusters	Cumulative Total of Clusters
HDC1	E1	4	4
HDC1	E2–E3	0	4
HDC1	E4	1	5
HDC1	E5–E7	0	5
HDC1	E8	2	7
HDC1/HDC2	E9–E10	0	7
HDC2	E11	2	9
HDC2	E12	1	10
HDC2/HDC3	E13–E21	0	10
HDC3	E22	2	12
HDC3	E23–E26	0	12
HDC4	E27	2	14
HDC4	E28–E34	0	14

**Fig 3:**
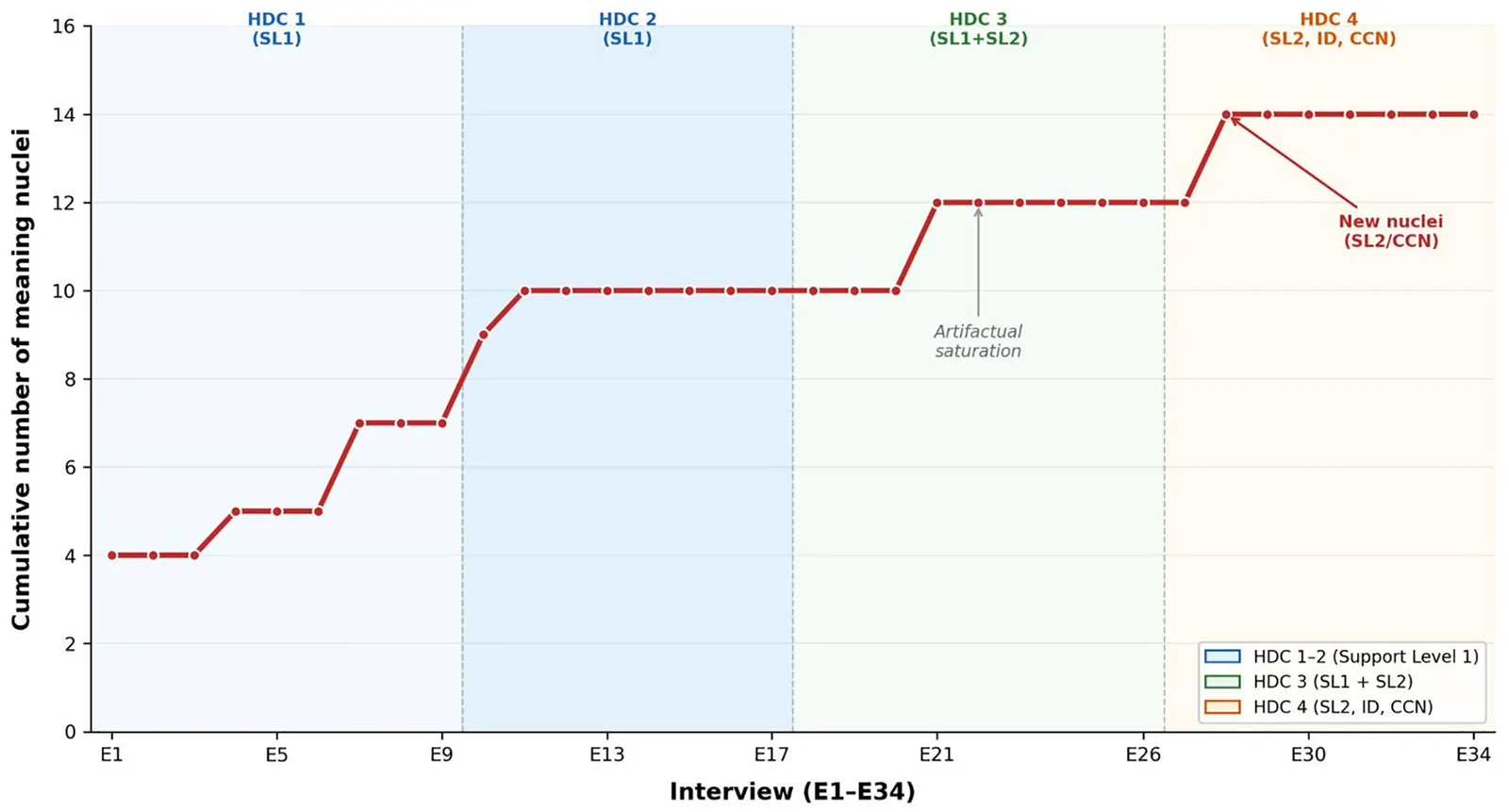
Cumulative category curve.

The saturation curve (Fig 3) shows a plateau that is interrupted by the emergence of new clusters in HDC4, indicating that the prior saturation was artifactual and restricted to a sampling subgroup, a finding that supported the decision to actively recruit participants for HDC4.

There were four HDCs: the first and third each included 9 participants; the second and fourth each included 8. The first and second HDCs included 17 women at support level 1 (E1–E17). The third (E18–E26) comprised 5 women at support level 1 and 4 at support level 2. The fourth included 8 women at support level 2 (E27–E34), of whom 4 had intellectual disabilities and 3 had complex communication needs (CCN) and were users of augmentative and alternative communication (AAC) (Figs 1 and 3). Observations regarding non-verbal and contextual aspects were recorded in the field journal.

### Validation of findings

Following the organization of results, findings were validated with 17 of the 34 participants, including 3 at support level 2, one of whom had CCN. The remaining participants did not respond to the invitation or declined participation in this phase. No participant requested withdrawal of their statements from the final material.

### Ethical aspects

The project was approved by the Research Ethics Committee of the University of Fortaleza, under opinion No. 6.205.069. All participants received, read, and agreed to the Informed Consent Form (ICF) in digital format prior to any data collection. In the case of participants at support level 2 with or without intellectual disability, considered legally incapable, the ICF was signed by the legal guardian. Adapted Assent Forms (e.g., simplified language, assisted reading, visual resources) were used to ensure that these participants could assent to and understand the research. Confidentiality was ensured through participant coding (E1–E34). To further protect anonymity, participant characteristics are reported in S2 Table using age bands, macro-regions of residence, aggregated occupational categories, and grouped rare comorbidities, and participants’ ages are not attached to individual quotations in the text or to individual codes in the figures.

### Participant characteristics

Thirty-four women with a formal diagnosis of ASD participated, with a mean age of 32.18 ± 7.19 years. Twenty-two had support level 1 and 12 had support level 2; three were non-speaking AAC users and four had some degree of intellectual disability. The mean interval between the last delivery and the interview was 2.86 ± 1.80 years (range: 3 months to 7 years).

Twenty-five participants were primiparous, eight had had two deliveries, and one had had three. Nineteen had given birth only vaginally, nine only by cesarean section, and six had experienced both modes of birth. Twenty-two received intrapartum care in public facilities of the Unified Health System (one of them accompanied by a privately hired doula) and 12 in the private sector. Twenty-six resided in state capitals, classified as urban residence, and eight in interior municipalities, classified as rural residence.

**Fig 4:**
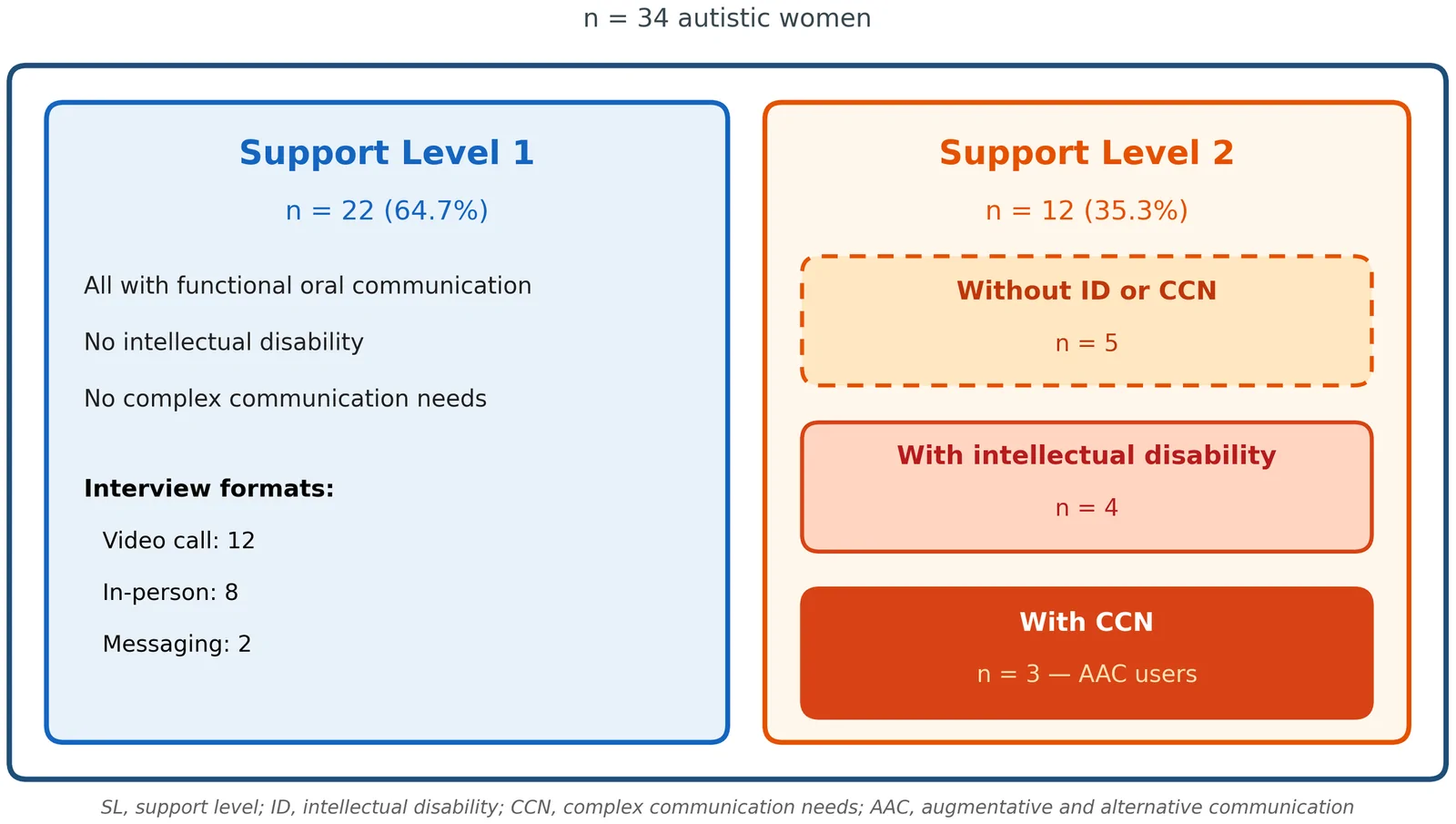
Participant profiles by support level, intellectual disability, and communication needs.

Regarding relationship status, 13 lived with a partner; 16 were single, of whom eight were in a non-cohabiting relationship; and five were divorced. Regarding sexual orientation, 13 identified as heterosexual, 11 as homosexual, five as bisexual, and three as pansexual; two preferred not to disclose. All five Brazilian macro-regions were represented: 16 participants from the Northeast, eight from the Southeast, six from the Central-West, three from the South, and one from the North. Regarding race/ethnicity, four identified as Black, two as Brown (mixed-race), and 28 as White. Regarding educational level, six had completed primary education, 14 had completed secondary education (two of whom were enrolled in undergraduate programs), nine held undergraduate degrees, and five held postgraduate qualifications. Detailed sociodemographic and clinical characteristics are presented in S2 Table. Fig 4 illustrates the variability of profiles according to support level, presence of intellectual disability, and CCN.

## Results

From the analysis, three central categories were constructed: (1) communicational demands of autistic women and their non-recognition; (2) communication barriers and institutional failures; and (3) experiences of respectful and effective communication. The internal organization of each category follows the meaning clusters described in the coding tree (S3 Table).

### Category 1: Communicational demands of autistic women and their non-recognition

This category articulates four meaning clusters: characteristics of autistic communicational and sensory processing, including a non-linear pain profile; the need for structured and predictable information; extreme episodes of overload, encompassing dissociative amnesia, meltdown and shutdown, and dissociation as a coping strategy followed by post-overload exhaustion; and the failure of professionals to recognize non-speaking communication as communication (S3 Table).

Across the four circles, participants described difficulty recognizing and naming emotions and bodily sensations, an experience many of them referred to through the diagnostic label of alexithymia. When professionals did not recognize this characteristic, clinical guidance premised on the spontaneous self-identification of symptoms became inoperative, producing care gaps with direct clinical risk:

*“I have always had this issue of not knowing exactly what I am feeling. I learned to rationalize. So, if I am irritated and having more difficulty processing things, I stop and think: ‘When did I last eat? Wow, I ate at 6 o’clock and it’s already noon! This must be hunger.’ But to know that it is hunger the way other people do, I simply don’t know.” (E5, SL1)*

*“Neurotypicals don’t understand. There’s no way they can understand. But when they ask ‘are you feeling such and such?’ or say ‘when you feel such and such, go to the hospital,’ well, that’s not how it works, because we can’t even properly identify that thing. I tried to explain this and the response I got was ‘then learn to know your own body.’ After that, I gave up trying to explain. It’s like talking to the walls.” (E8, SL1)*

This difficulty was not narrated as restricted to the pregnancy-postpartum period. Participants who constructed their subjectivity in contexts governed by neurotypical standards described having internalized the notion that their communicational difficulties stemmed from a personal failing, which translated into self-blame:

*“I have some difficulty even expressing what I am feeling [...] I think it’s me who can’t say everything I feel.” (E1, SL1)*

*“I think I was partly to blame. You’re talking to me. You can tell that I have difficulty speaking. [...] So I think they [the professionals] tried [to explain warning signs], but I have this difficulty understanding and talking about what I am feeling.” (E9, SL1)*

Sensory hypersensitivity crossed the accounts of participants at both support levels and appeared as a demand that the care environment systematically failed to accommodate. Rather than eliciting supportive responses, sensory overload was met with incomprehension or, as developed in the second category, with violent communication:

*“I was always hypersensitive. During pregnancy, I kept it under control because I knew how. But at the hospital... things got really bad. I fell apart completely. The smell, the sounds, everything irritated me. I had dreamed of a humanized birth, but it was an absolute hell.” (E12, SL1)*

*“Now, I love my baby. But when he was inside me, I hated him. Now I know I didn’t hate him, but his movements inside me were unbearable. I thought I had gone mad. [...] I tried to pierce my belly with scissors and another time with a knitting needle. [...] I didn’t want to die, I just wanted that hell to end [...] Every movement of his caused pain throughout my whole body and drove me to the edge of my sanity. I know I couldn’t explain it, but I was already losing my mind.” (E24, SL2)*

E24 also has Sensory Processing Disorder, diagnosed during pregnancy following hospitalization due to non-suicidal self-injury.

Closely linked to sensory processing, a non-linear pain profile crossed several accounts: hypoalgesia and hyperalgesia coexisting in the same trajectory, and a systematic mismatch between the outward expression of pain and its intensity, a mismatch that professionals read as absence of pain or as fabrication:

*“I have always been very resistant to pain, and my obstetrician knows it, because she is also my gynecologist. She used to say I would handle the cesarean easily. But that is not what happened. I received the spinal anesthesia, they laid me down and started working. I said I was feeling it. They sat me up and anesthetized me again. But I kept feeling it. I am not crazy, I felt pain. But both the obstetrician and the anesthesiologist said it was in my head, that I could not be feeling anything. But yes, I felt it, and it was a lot of pain. I felt pain through the entire cesarean.” (E6, SL1)*

*“I went through both experiences. My first was a vaginal birth and the second a cesarean. With the first, I arrived at the emergency department in a lot of pain. But the triage nurse did not believe me and gave me a green wristband, because of the risk classification, right? Red gets seen right away. Green waits. I kept saying I was in a lot of pain, but she told me I did not look like I was in pain. I gave birth in the hospital reception area. When my son started to be born I sat down on the floor, but I could not shout that he was coming. I lost my voice. I froze. It was a woman who was waiting there who shouted that a child was being born. Then everyone came running.” (E15, SL1)*

The need for predictability constituted a central demand. Even participants without complex communication needs described difficulty organizing their thoughts for oral communication during periods of overload, and reported that disruptions of predictability generated intense anxiety:

*“Even before diagnosis I always functioned on the basis of organization, predictability, and noting down details, and I always made this clear when consulting any physician, so they would know how I function and what to expect from me.” (E1, SL1)*

*“I ab-so-lute-ly NEED this kind of preview of how things are going to be. Otherwise I simply cannot function.” (E10, SL1)*

When these demands went unmet, the narratives converged on extreme episodes. Accounts of meltdown and shutdown during pregnancy, childbirth, and the postpartum period recurred across the circles:

*“It’s not something simple where I say: ‘oh, this bothers me, how annoying.’ No! I go into crisis! So much so that I had both meltdowns and shutdowns during pregnancy.” (E10, SL1)*

Dissociation and peripartum memory loss were likewise recurrent: participants across circles described “blackouts” and “blurry” memories of labor and birth, consistent with a dissociative response to an acute traumatic event:

*“I only have flashes of memory. I don’t remember much at all, exactly. It’s my husband who tells me what happened. I simply don’t remember.” (E4, SL1)*

Beyond involuntary memory loss, dissociation also appeared as a way of getting through overload, followed by a profound exhaustion that could itself be misread as a psychiatric condition:

*“I was exhausted. And it was not even the birth itself, because I could feel the contractions, but the pain was the least of my problems. We were supposed to be in individual rooms, but the hospital was full, so there were three women in the same room. One of them had music playing on her phone, and there was construction work going on in the hospital, that hammering all the time. It was too much noise at once. And people kept coming in and out of the room to use the bathroom, because the other room’s bathroom was closed off. That coming and going was making me feel very bad, and I was about to have a shutdown. So I went off to ‘partolândia’ [birth-la-la-land], as we say. I switched off. I blacked out. I have no memory of what happened. And I stayed that way for days. I could not breastfeed my daughter. They said it was postpartum depression, but it was not. My psychiatrist agreed with me afterwards that it was really exhaustion, that I did not meet criteria for depression.” (E17, SL1)*

For non-speaking participants, the demand was more elementary: that their communication be recognized as communication. These participants indicated that professionals tended to ignore non-speaking patients, describing a sense of abandonment. One reported that no explanation was provided about the stages of intrapartum care, a finding corroborated by another AAC user, who wrote: *“I felt like an object. I was taken from here to there. No one explains anything.” (E32, SL2)*

### Category 2: Communication barriers and institutional failures

This category articulates seven meaning clusters: professionals’ unfamiliarity with autism in adult women, including the scientific knowledge gap identified by participants themselves; disregard for the woman’s knowledge of her own body; the dilemma of diagnosis disclosure and its consequences; rupture of trust and institutional violence, including disrespectful and dehumanizing treatment and participants’ own naming of obstetric violence; care lapses attributable to communication failure; the ambivalent role of the companion; and camouflaging with the consequent misreading of atypical presentations (S3 Table).

The narratives converge on a dissonance between the communicational demands described in the first category and current care practice. Unfamiliarity with the manifestations of autism in adult women (several participants were still undiagnosed during pregnancy), combined with disregard for women’s self-knowledge, resulted in the minimization of complaints and the invalidation of suffering:

*“I mentioned it during prenatal care [the hypersensitivity to fetal movements] and they said it was normal, but I tried to explain that it wasn’t, because I was having absence seizures, and they thought I was exaggerating. At the time, I didn’t have a diagnosis, right? But they just kept saying it was normal, normal.” (E23, SL2)*

Some participants themselves traced this unfamiliarity to its upstream cause: a scientific gap, the absence of research on autistic bodies in obstetric care:

*“Look, I kept asking myself: has no one ever researched this? Later I searched the internet and talked with other women. Many others had been through the same thing [feeling the surgery despite anesthesia]. But you know what it is? They assume everybody’s body is neurotypical. Now answer me this: if the head commands the body, and the head works differently, why would the body respond the same?” (E14, SL1)*

Anticipating disbelief, participants also described a calculation about whether to disclose the diagnosis at all: a dilemma informed by earlier experiences of retaliation and resolved, at times, through concealment until disclosure became unavoidable:

*“I already had the diagnosis, but yes, I was afraid to tell. [...] when I got the diagnosis I took the report to the company to request some accommodations. And it was not even much: being able to use ear defenders, reducing the visual stimuli in the room [...] but what happened is that they started treating me as incapable. [...] Then came the dismissal. So that stayed in my head: if I say I am autistic, they will not give me autonomy. And I was right. I only told at the hospital after the birth, to explain why I was having so much difficulty with breastfeeding. Well... the nursing technicians seemed afraid to let me hold my own son. And I did keep thinking: which is worse: having no accommodation because you did not disclose the diagnosis, or disclosing and being treated as an invalid?” (E13, SL1)*

The camouflaging that many participants had refined over a lifetime compounded the invalidation: an outward appearance of regulation led teams to underestimate pain and urgency, while atypical presentations were read as exaggeration or melodrama rather than as clinical information:

*“I mask very well. Or not, I do not even know. Because my poker face is the same for everything. I am dying inside and the person thinks everything is fine. And that is a problem, because no one ever believes me when I say I am feeling something.” (E15, SL1)*

In its most extreme form, this misreading escalated into disrespectful treatment and violence, which participants themselves named as such:

*“At every hospital it was the same. One of them said that if they were to induce labor for every woman who feels discomfort, every baby would be born premature. Another one started treating me very badly and was extremely rude. She even said that God punishes women who speak about their child the way I was speaking. I tried to explain that it was unbearable, but they thought I was being melodramatic. The nurses looked at me as if I were a criminal.” (E24, SL2)*

*“[...] no one should have to pay for a respectful birth, but I hired a team. I asked for references about whether they were truly humanized, and all the references were excellent. As it turned out, that was no guarantee of anything. My SPD exploded during labor. I was screaming: ‘Don’t touch me.’ It made no difference. You know that whole thing about autonomy? Well, that didn’t happen. [...] The entire team seemed frustrated. I overheard the physician whispering to the doula that she had thought I would be great but hadn’t expected me to be so much work. Those words, at that moment, in that situation, felt like a tremendous act of violence against me.” (E11, SL1)*

The naming of violence was not restricted to speaking participants. Asked for a word that recalled her birth, one non-speaking participant pointed to two pictograms: “pain” and “violence.” Asked why, she pressed the pictograms for “doctor” and “anger,” and then typed: “my fault?” *(E27, SL2; AAC-mediated interview, field journal)*

Participants connected these communicational failures to concrete repercussions in their care trajectories:

*“I froze completely during labor. I think he wasn’t born vaginally because I was completely frozen, and then the nurses kept touching me. It was sending me into a panic.” (E2, SL1)*

*“I explained that I wouldn’t be able to identify a headache, but he kept saying that if my blood pressure went up I should go to the maternity ward and that I should take my blood pressure if I felt something. As it turned out, I had a seizure at home. That was the eclampsia.” (E7, SL1)*

Continuity of care and the presence of a companion able to mediate communication appeared as fundamental needs. When the companion became the primary recipient of professional communication, however, mediation converted into substitution, and the woman was suppressed as interlocutor:

*“[The healthcare professionals] didn’t even talk to you, they only talked to my mother, and I felt terrible about that. [...] I overheard them whispering. They thought I wasn’t noticing, and it only increased my distress.” (E23, SL2)*

For participants with CCN, this process of communicational exclusion compromised emotional wellbeing and dignity. One participant, who communicates through writing and pictograms, when asked to whom professionals addressed themselves during prenatal care and labor, pointed to the pictogram for “mother” and wrote: *“they didn’t talk to me.”* She then wrote: *“I invisible.”* When asked how she felt, she was unable to write, but pointed to the pictogram meaning “sad” *(E29, SL2)*. Another participant, whose pregnancy resulted from a relationship with a young man who was also autistic and had CCN, expressed via AAC: *“They felt sorry for me. [...] They said: ‘it’s cruel to get her pregnant.’ I felt bad.” (E27, SL2)*

### Category 3: Experiences of respectful and effective communication

This category articulates three meaning clusters: successful communicational adaptations; the role of multiprofessional articulation; and the distribution of the burden of adaptation, that is, who produced each adaptation and at what cost, encompassing the woman’s own initiative, the confrontation sometimes required to obtain adaptations, and dependence on economic resources or personal networks (S3 Table).

Despite the predominance of accounts of barriers, experiences of successful communication demonstrated that care organization can be adapted:

*“For me, for example, waiting was a problem, so I would already say that I can’t handle it, it disorganizes me, I get very irritated. So he [the prenatal physician] would schedule the exact time and, at the exact time, they would call me. Not before, not after.” (E22, SL2)*

*“She [the doula] helped me a lot with the contact with the nurses, because the nurses made me very anxious [...] I arranged with her that I would speak to her, and depending on the signals, she would pass things along to the nursing staff. She was very respectful.” (E3, SL1)*

*“The physician who admitted me [...] said right away that I had sensory processing disorder. [...] Later the psychiatrist confirmed it. [...] The occupational therapist did some therapies that improved the discomfort a little. [...] But that little improvement was fundamental for me to hold on a little longer until it was time for my delivery. [...] But I already started to feel better just from the physician saying that if I had any questions I could look for her. I would write down my questions and whenever she was on call I would go ask her because she explained things in a way I could understand.” (E24, SL2)*

*“The nurse stapled a paper with a circle onto my prenatal card. Inside the circle were the weeks, and she would color in the weeks using different colors as things were happening. That calmed me down a lot.” (E30, SL2)*

Even when adaptation was eventually obtained, participants described its price. For E6, being able to eat after her cesarean required first open confrontation, and then a personal connection inside the service:

*“As soon as [my son] was born, they put something in my IV to make me sleep, because I was screaming in pain. The anesthesiologist told me, and I shouted that I did not want to sleep, I wanted the pain to stop, but I wanted to see my son. He sedated me anyway. When I woke up I was hypersensitive. Everything irritated me. My tolerance threshold was at rock bottom. Then my food selectivity came back with full force. I could not eat anything mushy, and the food they brought was mashed potatoes with meat in sauce. I vomited. I said I could not eat that, and they treated it as fussiness. I had to deliver a whole treatise on food selectivity, scream, kick up a fuss, for them to let food be brought in from outside, otherwise I would have gone hungry.” (E6, SL1)*

*“What saved me was that my partner ran into a colleague of his who works at the hospital. This colleague went to talk with the nutritionist, and they allowed my partner to go out and bring food from outside. Because under the hospital’s rules, even though it was private, that was not allowed.” (E6, SL1)*

Institutional initiative was not entirely absent, but it surfaced in a single account and, even then, contingent on the disclosure of the diagnosis and on the happenstance of the resource existing in that service. It is also the only account in which disclosing the diagnosis was met with a resourced institutional response:

*“When I said I was autistic, the obstetrician asked for the hospital’s occupational therapist to come. She did some activities with me that regulated me quite a bit. If every hospital had an occupational therapist, that alone would already help a lot.” (E18, SL1)*

Read together, these experiences reveal a consistent pattern in who bore the burden of adaptation. Successful arrangements rarely originated from institutional protocol: they depended on the woman’s own anticipatory work of instructing professionals about her functioning; on exhausting confrontation until a demand was met; on privately contracted resources, which, as the account of E11 in the previous category shows, offered no guarantee; on personal networks and chance connections inside services, as in E6’s account; or on the initiative of individual professionals, such as the multiprofessional articulation described by E24 and the nurse who built E30’s visual timeline. Adaptation mobilized by the service itself appeared in a single account, dependent on disclosure and on the availability of the resource: an exception that underscores the rule.

## Discussion

Analysis of the narratives indicates that responsibility for effective communication has been disproportionately attributed to patients, an inversion that participants experienced as a patient safety problem. The timely identification of warning signs depends on active inquiry, directed questions, comprehension checks, and the construction of an accessible communicational environment: responsibilities that rest with the healthcare system, not with the patient. In the literature, failures in these practices (including insufficient information, inadequate shared decision-making, and poor response to reported concerns) are associated with a higher likelihood of avoidable cesarean sections, diagnostic delays, and worse mental health outcomes [18,30,31]. The present qualitative design does not permit causal claims of this kind; what the narratives document is how, in the participants’ experience, communication failures and adverse events appeared intertwined.

The specific communicational demands identified in this study (alexithymia, need for predictability, and sensory hypersensitivity) are not individual idiosyncrasies, but manifestations of autistic neurodevelopment [4,5,32]. Difficulty verbalizing sensations should not be interpreted as an absence of symptoms, but as an indication that professionals must employ alternative communication tools and more structured questioning. Ignoring this need, as illustrated by E8’s account (“then learn to know your own body”), constitutes an attitudinal barrier that invalidates the patient’s experience, a dynamic that, in E7’s account, preceded an eclamptic seizure at home after guidance premised on the self-identification of warning signs she had explicitly stated she could not recognize.

Sensory overload in hospital environments, with intense lighting, noise, smells, movement, and unexpected or unconsented touch, is not mere subjective discomfort, but a trigger for acute stress responses that can precipitate emotional dysregulation (meltdown), withdrawal (shutdown), and self-injurious behaviors, as experienced by E24 [5,33]. This picture is aggravated when Sensory Processing Disorder (SPD) coexists, characterized by difficulty modulating and responding to sensory information, and may manifest as hyper-reactivity (exaggerated and aversive responses to stimuli such as light, sound, touch, and smell), hypo-sensitivity, or stimulus seeking/avoidance, with significant functional repercussions [6,34]. In hyper-reactivity, ordinary stimuli may be perceived as aversive and painful, with a direct impact on physical and mental health [35–37]. During pregnancy, in the absence of sensory and communicational adaptations, bodily and environmental changes can amplify pre-existing sensitivities and render care experiences (touch, noisy environments, procedures) more aversive [6,37]. Such responses may be erroneously interpreted by the healthcare team as “anxiety,” “exaggeration,” or “lack of cooperation,” rather than being recognized as manifestations of sensory overload [37].

The presence of functional speech does not guarantee effective communication. Even among verbally fluent autistic individuals, limitations persist in the pragmatic domain (the use of language, gesture, prosody, and context to construct meaning), and communication barriers may arise from interactions with poorly adapted environments and interlocutors [38,39]. Among individuals with lower support needs, compensatory strategies and camouflaging are common (scripted conversations, imitation, monitoring of eye contact, expressions, and tone of voice), which reduce the visibility of difficulties at the cost of continuous effort [40,41]. During periods of sensory overload and energy depletion, hyper-reactivity tends to intensify, and self-regulation may collapse, with withdrawal, reduced functioning, and temporary loss of communicative skills, a scenario in which compensatory mechanisms can no longer sustain communication [36,42].

For participants with CCN, the barrier is even more explicit, as evidenced by the participant who described herself as “invisible” when professionals addressed themselves exclusively to her mother. CCN refers to situations in which an individual cannot use speech alone to meet the functional communicational needs of daily life, requiring the support of communication partners and/or supplementary or alternative communication resources [43]. In clinical practice, this is operationalized through a repertoire of strategies ranging from unaided resources (gestures, agreed-upon signs, yes/no responses, pauses, key words) to low-technology AAC (paper, pen, letter/picture boards, choice cards, visual pain scales, consent scripts) and high-technology AAC (applications and speech-generating devices with access via touch, switches, or eye tracking), combined with direct language, free of jargon, active comprehension checks, and additional processing time [44]. The literature demonstrates that the provision of AAC tools, even low-technology ones, improves patient autonomy and satisfaction, but its implementation depends on the initiative and training of the care team, not of the patient [45–47]. Recognizing CCN, including communicational fluctuations under pain, stress, or overload, and providing, from the prenatal period onward, a communication plan (passport/preferences guide, pause signals, AAC options, explicit consent for touch) constitutes an accessibility intervention that should be an institutional responsibility [44–46].

The pattern identified in the third category (adaptations produced by the woman herself, by privately contracted resources, or by the initiative of individual professionals, and only exceptionally by the service itself) points to the structural layers that sustain the barriers. The first layer concerns professional education: the unfamiliarity that participants repeatedly encountered suggests that autism in adult women and communicational accessibility occupy a marginal place in professional training, a pattern consistent with the literature on the healthcare experiences of autistic adults [4,14,33]. The second concerns organizational culture: task-oriented routines, time pressure, and the normalization of disrespect documented in Brazilian obstetric care [18] leave little room for the anticipation, predictability, and comprehension checks that participants needed; professional unfamiliarity with these particularities aggravates the risk of institutional violence and compromises shared decision-making [15,39,48,49], to which women from socially marginalized groups are already less likely to have access [10]. The third layer concerns the architecture of the maternity-care system itself: the coexistence of a universal public system and a private sector produces stratified trajectories, yet barriers crossed both sectors in the narratives (participants who privately contracted “humanized” teams reported failures analogous to those described in public services), suggesting that the problem is not reducible to resource scarcity. Comparable dynamics have been described in other low- and middle-income countries: asymmetries in physician–patient decision-making about cesarean sections in Bangladesh [30] and the burden of postpartum post-traumatic stress documented in Ethiopia [50] indicate that communicational vulnerability compounds structural strain in maternity systems of these settings. The present findings add the specific configuration that autistic women face within such systems, in which power asymmetry deepens precisely when sensory overload compromises their communicational capacity and self-regulation [15].

Insecurity and inadequate communication can trigger neurophysiological threat responses, activating the fear-tension-pain cycle, with increased stress, sympathetic activation, and impairment of the neurohormonal dynamics of labor [50–52]. In this sense, inadequate communication and insecurity are not merely subjective experiences, but factors with potential direct impact on the physiology of labor [51].

The reports of peripartum memory “blackouts” and dissociation are consistent with responses to acute trauma and represent a risk factor for childbirth-related Post-Traumatic Stress Disorder (PTSD), a condition for which autistic women appear to exhibit increased vulnerability [35,36,50,53,54]. Recent evidence corroborates that episodes of intense overload during childbirth can be experienced as traumatic events when care does not provide predictability, robust consent, and sensory and communicational adaptations [6,48].

In contrast, the positive experiences reported in Category 3 provide evidence that care adaptation is both feasible and effective. Professionals who practice active listening, validate the patient’s experience, offer predictability, and adapt the environment and communication promote safety and wellbeing. The findings allow these practices to be translated into specific competencies for professional training and routine maternity care: structured, closed-ended symptom inquiry that does not presuppose the spontaneous verbal report of sensations; systematic comprehension checks, such as teach-back techniques; explicit requests for consent before any touch; sensory adaptation of care environments, including lighting, noise, and the number of people present; the routine offer of low-technology AAC resources; the construction, from prenatal care onward, of an individual communication plan documenting preferences, pause signals, and consent arrangements; and the guarantee of continuity of care and of the companion’s role as mediator rather than substitute. Strategies such as accessible birth plans, written information, use of visual supports, and explicit consent prior to touch do not constitute extraordinary adaptations, but rather the materialization of person-centered care and communicational accessibility [5,51,55,56]. Such practices are central elements of inclusive health systems aligned with policies for the removal of barriers for people with disabilities [4,56] and, as the findings of this study suggest, are fundamental to reversing the scenario of health inequities affecting this population.

### Strengths and limitations

The primary strength of this study lies in the deliberate inclusion of autistic women with different support levels and communicational needs, including participants with CCN and intellectual disabilities, profiles underrepresented in health research. The use of multiple interview formats ensured accessibility and broadened the representativeness of the sample. The researcher’s reflexivity, as a neurodivergent woman herself, was a facilitating factor for the establishment of trust and the elicitation of rich narratives. The credibility of the findings is sustained by the rigor of the analytical process, including independent coding with consensus meetings, peer debriefing between research groups, an audit trail, participant validation, and saturation monitoring across the four HDCs.

Several limitations must be acknowledged. The analysis is based on retrospective self-reports, subject to memory biases, although peripartum memory loss itself was constructed as a relevant finding. Recruitment predominantly via social media and participant referral favors women engaged with advocacy networks and with access to technology, which limits transferability: the perspectives of autistic women disconnected from such networks, with later or no diagnosis, or with restricted connectivity are likely underrepresented. The predominance of White participants (82%) and of women at support level 1 confirms this selection bias and points to the need for studies with greater participation of Black, Indigenous, low-income, and higher-support-need autistic women. The different interview modalities (video call, messaging, in-person, AAC-mediated) produce qualitatively different data in terms of depth, spontaneity, and opportunities for non-verbal observation; this heterogeneity was monitored reflexively but cannot be eliminated, and was the deliberate cost of broadening participation. The single-interviewer design, while central to establishing trust, concentrates elicitation in one positionality; this was mitigated, but not neutralized, by the reflexive journal and by peer debriefing. Finally, as a qualitative and retrospective study, the findings represent participants’ lived experiences and interpretations; they do not constitute direct evidence of causal mechanisms linking communication barriers to clinical outcomes, and the accounts were not triangulated with medical records.

## Conclusion

This study analyzed, from the narratives of 34 autistic women, communication barriers experienced in perinatal care in Brazil. The narratives indicate that these barriers are experienced as systemic rather than individual: they arise less from characteristics of autistic functioning than from the inability of services to offer flexible, informed, and person-centered communication. The accounts of respectful communication demonstrate that adaptation is feasible and that its burden currently falls on the women themselves rather than on institutions. Training healthcare professionals in accessible communication, implementing communication plans from prenatal care onward, and strengthening the companion’s role as an active mediator constitute low-cost strategies whose implementation and evaluation should be prioritized. Future research should measure the prevalence of the barriers identified here, test the efficacy of educational interventions, and deepen the intersectional analysis by cross-referencing data with race/ethnicity and socioeconomic class.

## Supporting information

S1 Checklist COREQ (COnsolidated criteria for REporting Qualitative research) checklist. (DOCX)

S2 Table Sociodemographic and clinical characteristics of the participants (n = 34). (XLSX)

S3 Table Coding tree: categories, meaning clusters, and operational definitions. (DOCX)
