# Supplementary material for: Communication barriers in perinatal care for autistic women: A qualitative study using the hermeneutic-dialectic circle in Brazil
Source: PLoS One. 2026 Sep 16;21(9):e0358429. doi: 10.1371/journal.pone.0358429 (PMC13581197; doi:10.1371/journal.pone.0358429)
Supplement: Supplementary file 1 [file pone.0358429.s001.docx]

**S1 Checklist.** COREQ (COnsolidated criteria for REporting Qualitative research) 32-item checklist.

*Tong A, Sainsbury P, Craig J. Consolidated criteria for reporting qualitative research (COREQ): a 32-item checklist for interviews and focus groups. Int J Qual Health Care. 2007;19(6):349–357.*

Manuscript: Communication barriers in perinatal care for autistic women: a qualitative study using the Hermeneutic-Dialectic Circle in Brazil (PONE-D-26-13455). Locations refer to the sections of the revised manuscript.

**Domain 1: Research team and reflexivity**

| **No.** | **Item** | **Guide question/description** | **Reported in** |
| --- | --- | --- | --- |
| 1 | Interviewer/facilitator | *Which author/s conducted the interview?* | A single researcher (the first author) conducted all 34 interviews. Methods – Data collection. |
| 2 | Credentials | *What were the researcher’s credentials?* | Obstetrician; doctoral-level physician (MD, PhD). Methods – Data collection. |
| 3 | Occupation | *What was their occupation at the time of the study?* | University professor affiliated with a graduate program. Methods – Data collection. |
| 4 | Gender | *Was the researcher male or female?* | Female. Methods – Data collection. |
| 5 | Experience and training | *What experience or training did the researcher have?* | Experience in qualitative research with autistic individuals (refs 24,25); the principal researcher additionally trained the two coding analysts in NVivo and in the coding process. Methods – Data collection; Data analysis. |
| 6 | Relationship established | *Was a relationship established prior to study commencement?* | No. The researcher had no prior personal or professional relationship with any participant. Methods – Data collection. |
| 7 | Participant knowledge of the interviewer | *What did the participants know about the researcher?* | At the start of each meeting the interviewer introduced herself, declaring her status as a physician, researcher, and neurodivergent woman, and clarified the research motivations. Methods – Data collection. |
| 8 | Interviewer characteristics | *What characteristics were reported about the interviewer/facilitator?* | Neurodivergent woman; positionality and potential biases addressed through a reflexive journal maintained throughout the study and discussed as a limitation. Methods – Data collection; Discussion – Strengths and limitations. |

**Domain 2: Study design**

| **No.** | **Item** | **Guide question/description** | **Reported in** |
| --- | --- | --- | --- |
| 9 | Methodological orientation and theory | *What methodological orientation was stated to underpin the study?* | Constructivist paradigm; Hermeneutic-Dialectic Circle (Lincoln and Guba), with the constant comparative method as the operational analytical strategy; the social model of disability and the neurodiversity paradigm as interpretive lenses. Methods – Study design and theoretical-methodological framework; Data analysis. |
| 10 | Sampling | *How were participants selected?* | Convenience sampling, supplemented by active (purposive) recruitment via participant referral to include women at support level 2. Methods – Participants and recruitment. |
| 11 | Method of approach | *How were participants approached?* | Invitation disseminated on social media (Facebook, Instagram) and in WhatsApp groups of autism associations and collectives; enrollment via electronic form. Methods – Participants and recruitment. |
| 12 | Sample size | *How many participants were in the study?* | 34. Methods – Participant characteristics; Fig 1. |
| 13 | Non-participation | *How many people refused to participate or dropped out? Reasons?* | Of 68 women who accessed the form, 26 did not agree to the qualitative phase and 16 withdrew before the interview; reasons for withdrawal were not systematically collected. Methods – Participants and recruitment; Fig 1. |
| 14 | Setting of data collection | *Where was the data collected?* | Video call (n = 15), real-time audio and text messaging (n = 6), and in person at a location chosen by the participant (n = 13). Methods – Data collection. |
| 15 | Presence of non-participants | *Was anyone else present besides the participants and researchers?* | In three interviews with support level 2 participants with intellectual disability or complex communication needs, a facilitator was present: the legal guardian in one interview and, in two, therapeutic companions who assisted in the application of AAC. Methods – Data collection. |
| 16 | Description of sample | *What are the important characteristics of the sample?* | Age, support level, intellectual disability, complex communication needs, parity, mode of birth, care sector, urban/rural residence, relationship status, sexual orientation, macro-region, race/ethnicity, education. Methods – Participant characteristics; S2 Table; Fig 4. |
| 17 | Interview guide | *Were questions, prompts, guides provided by the authors? Was it pilot tested?* | Semi-structured guide developed from the literature; piloted with an advisory committee of four autistic women external to the sample; adapted for each interview as proposed by the HDC. Methods – Data collection. |
| 18 | Repeat interviews | *Were repeat interviews carried out?* | Nine participants were contacted a second time for additional clarification. Methods – Data collection. |
| 19 | Audio/visual recording | *Did the research use audio or visual recording to collect the data?* | Interviews were recorded in audio and/or video, with consent; text-based and AAC-mediated interviews were documented in the participants’ own written productions and pictogram selections. Methods – Data collection; Data analysis. |
| 20 | Field notes | *Were field notes made during and/or after the interview?* | Yes; a field journal (contextual and non-verbal observations) and a reflexive journal were maintained and integrated into the analytical corpus. Methods – Data collection; Data analysis. |
| 21 | Duration | *What was the duration of the interviews?* | Mean 40 minutes (range 32–63). Methods – Data collection. |
| 22 | Data saturation | *Was data saturation discussed?* | Yes; monitored with an operational criterion (saturation matrix and cumulative category curve), including the identification of artifactual saturation restricted to a sampling subgroup. Methods – Sample adequacy; Table 1; Fig 3. |
| 23 | Transcripts returned | *Were transcripts returned to participants for comment and/or correction?* | Transcripts were not returned; instead, the organized findings were validated with participants (see item 28). Methods – Validation of findings. |

**Domain 3: Analysis and findings**

| **No.** | **Item** | **Guide question/description** | **Reported in** |
| --- | --- | --- | --- |
| 24 | Number of data coders | *How many data coders coded the data?* | Two trained analysts coded independently; a third researcher mediated consensus meetings; persistent disagreements were taken to inter-group methodological oversight meetings (peer debriefing). Methods – Data analysis. |
| 25 | Description of the coding tree | *Did authors provide a description of the coding tree?* | Yes; the full coding tree, with categories, fourteen meaning clusters, and operational definitions, is provided as S3 Table. Methods – Data analysis; S3 Table. |
| 26 | Derivation of themes | *Were themes identified in advance or derived from the data?* | Categories were constructed from the data through the iterative cycles of the HDC (constant comparison, successive consensuses, cycle syntheses), in dialectical confrontation with the literature. Methods – Data analysis. |
| 27 | Software | *What software, if applicable, was used to manage the data?* | NVivo, version 12. Methods – Data analysis. |
| 28 | Participant checking | *Did participants provide feedback on the findings?* | Yes; findings were validated with 17 of the 34 participants, including three at support level 2 (one with complex communication needs); no participant requested withdrawal of statements. Methods – Validation of findings. |
| 29 | Quotations presented | *Were participant quotations presented to illustrate the themes/findings?* | Yes; each meaning cluster is illustrated with quotations from multiple participants, identified by code and support level (25 of 34 participants quoted). Results. |
| 30 | Data and findings consistent | *Was there consistency between the data presented and the findings?* | Yes; interpretive claims are anchored in the quoted data, the coding tree (S3 Table), and the saturation matrix (Table 1). Results; Discussion. |
| 31 | Clarity of major themes | *Were major themes clearly presented in the findings?* | Yes; three central categories, each opening with a paragraph naming its constituent meaning clusters. Results. |
| 32 | Clarity of minor themes | *Is there a description of diverse cases or discussion of minor themes?* | Yes; divergent and exceptional cases are reported, including the single account of institutionally initiated adaptation and the near-absence of this pattern in the corpus, and positive experiences contrasting with the predominant barriers. Results – Category 3; S3 Table. |
