# Supplementary material for: Communication barriers in perinatal care for autistic women: A qualitative study using the hermeneutic-dialectic circle in Brazil
Source: PLoS One. 2026 Sep 16;21(9):e0358429. doi: 10.1371/journal.pone.0358429 (PMC13581197; doi:10.1371/journal.pone.0358429)
Supplement: Supplementary file 2 [file pone.0358429.s003.docx]

**S3 Table. Coding tree: categories, meaning clusters, and operational definitions**

**Category 1: Communicational demands of autistic women and their non-recognition**

| **Code** | **Meaning cluster / node** | **Operational definition** |
| --- | --- | --- |
| 1.1 | **Characteristics of autistic processing** |  |
| 1.1.1 | Characteristics of autistic communicational processing | Descriptions of how the participant formulates, organizes, and conveys verbal content, including difficulty with assertion and channel preference |
| 1.1.2 | Sensory hypersensitivity complicating communication | Alterations in sensory processing that interfere with the ability to communicate or to sustain interaction |
| 1.1.3 | Non-linear pain profile | Coexistence of hypoalgesia and hyperalgesia in the same participant; lack of correspondence between outward expression and pain intensity |
| 1.2 | **Need for structured and predictable information** | Demands for anticipation, planning, and informational detail, and the effects of their disruption |
| 1.3 | **Aggravations and extreme episodes** |  |
| 1.3.1 | Intrapartum dissociative amnesia | Reports of absence, fragmentation, or blurring of memory of segments of labor or birth, including reported loss of consciousness |
| 1.3.2 | Shutdown and meltdown in the experience of pregnancy and childbirth | Occurrence of autistic crises, with or without motor manifestation, during pregnancy, childbirth, or the postpartum period |
| 1.3.3 | Dissociation as coping and post-overload fatigue | Deliberate or involuntary use of perceptual distancing to get through the process, and exhaustion following sensory overload |
| 1.4 | **Professionals’ failure to understand non-oral forms of communication** | Professional failure to read bodily, behavioral, and paraverbal signals as communicative content |

**Category 2: Communication barriers and institutional failures**

| **Code** | **Meaning cluster / node** | **Operational definition** |
| --- | --- | --- |
| 2.1 | **Unfamiliarity with autism in adult women** | Stereotypes, absence of diagnostic hypothesis, late-diagnosis trajectories, and barriers to accessing assessment |
| 2.1.1 | Scientific knowledge gap identified by the participant | Identification, by the interviewee herself, of the absence of research or evidence on issues that affected her care |
| 2.2 | **Disregard for the woman’s knowledge of her own body** | Bodily or pain reports communicated and not incorporated into clinical conduct; explicit refusals disrespected |
| 2.3 | **Dilemma and negative consequences of diagnosis disclosure** | Calculation about whether to disclose; acceptance or retaliation following disclosure of the diagnosis or of a need |
| 2.4 | **Rupture of trust in the therapeutic relationship and institutional violence** | Rupture of bond, consequent self-silencing, ideological bias of the professional |
| 2.4.1 | Disrespectful and dehumanizing treatment | Suppression of the woman as interlocutor, parallel communication, violation of environment and body |
| 2.4.2 | Naming of obstetric violence by the participant | Excerpts or communicative acts in which the interviewee herself identifies the event experienced as violence |
| 2.5 | **Care lapses resulting from the barriers** | Adverse outcomes, omissions, delays, and complications attributable to communicational or recognition failure |
| 2.6 | **Role of the advocate/companion** | Mediation by third parties, whether as effective support or as substitution of the woman as interlocutor |
| 2.7 | **Camouflaging and misreading of the presentation** | Outward appearance of regulation, calm, or normality inducing clinical underestimation; atypical manifestation read as simulation, exaggeration, or a psychiatric condition; cost of the masking effort |

**Category 3: Experiences of respectful and effective communication**

| **Code** | **Meaning cluster / node** | **Operational definition** |
| --- | --- | --- |
| 3.1 | **Successful communicational adaptations** | Concrete adjustments of channel, environment, flow, or procedure that produced a favorable effect |
| 3.2 | **Multiprofessional team** | Team arrangements, integration, and intersectoral articulation |
| 3.3 | **Distribution of the burden of adaptation** | Identification of who produced the adaptation and at what cost: the woman’s initiative, economic resources, personal network, institutional chance, or institutional initiative |
| 3.3.1 | Adaptation on the woman’s own initiative | Prior instruction of professionals, informational literacy, anticipatory preparation |
| 3.3.2 | Cost of the confrontation required to obtain the adaptation | Need to insist, argue, or actively refuse for the demand to be met |
| 3.3.3 | Adaptation dependent on economic resources | Private hiring, self-funded preconception planning, paid specialized consultancy |
| 3.3.4 | Adaptation dependent on personal network or institutional chance | Access obtained through family ties, a known professional, or a referral coincidence |
| 3.3.5 | Adaptation on institutional initiative | Adaptation offered by protocol, routine, or service initiative, without prior demand from the woman |

*Meaning clusters are groupings of meaning units expressing recurring interpretive patterns, constructed across the four Hermeneutic-Dialectic Circles. A single account of adaptation mobilized on institutional initiative (3.3.5) was identified in the corpus; the node registers the near-absence of institutionally initiated adaptations.*
